# Editorial: Digital Technology in Neurology: From Clinical Assessment to Neurorehabilitation

**DOI:** 10.3389/fneur.2020.614074

**Published:** 2021-01-21

**Authors:** Marcello Moccia, Francesco Brigo, Sabina Brennan, Simona Bonavita

**Affiliations:** ^1^Department of Neuroscience, University of Naples “Federico II”, Naples, Italy; ^2^Department of Neurology, Hospital of Merano, Azienda Sanitaria Dell'Alto Adige (ASDAA-SABES), Merano, Italy; ^3^Institute of Neuroscience, Trinity College Dublin, Dublin, Ireland; ^4^Department of Advanced Medical and Surgical Sciences, University of Campania “Luigi Vanvitelli”, Naples, Italy

**Keywords:** digital technology, multiple sclerosis, stroke, neurorehabilitation, neurology

Over the past decades, advances in digital technology have led to the introduction of electronic health (eHealth) applications (1). Considering that many patients with chronic and disabling neurological diseases have complex healthcare needs but difficulties in access (e.g., mobility restrictions), digital technology has become progressively used to improve delivery of healthcare services, clinical assessments, and data collection in research and clinical practice. Hereby, we aim to review the results presented in this special issue on the use of digital technology in neurology (2).

Telerehabilitation encompasses prevention, evaluation, assessment, intervention, monitoring, supervision, education, consultation, and coaching, and, as such, has been used to deliver different interventions, with the possibility to provide patients with real-time feedback on rehabilitation outcomes to improve engagement and, thus, promote neuroplasticity and functional recovery. In line with this, the systematic review by Matamala-Gomez et al. has highlighted an increasing interest in creating new telerehabilitation protocols for enhancing patients' engagement by promoting self-awareness, self-management, and motivation, and by providing emotional support; of note, positive results were generally seen by enhancing the behavioral, cognitive, and emotional dimensions of patient engagement. Wu et al. have investigated the impact brain-computer interface (BCI)-based training has on upper limb rehabilitation in subacute stroke patients, using functional connectivity MRI analysis. Briefly, the BCI-based training provided users with brain state-dependent sensory feedback via functional electrical stimulation, virtual reality environments, or robotic systems, and has determined reorganization of brain functional networks topology in subacute stroke patients, with increased coordination between the multi-sensory and motor-related cortex and the extrapyramidal system. Similarly, Li et al. used repetitive transcranial magnetic stimulation (rTMS) for cognitive rehabilitation in post-stroke cognitive impairment (PSCI) and showed both cognitive improvement following rTMS, and change in neural activity and functional connectivity in cognition-related regions on resting-state functional MRI (Li et al.). Patients' satisfaction with a technology-enabled rehabilitation program was investigated by Isernia et al. in people with different central nervous system diseases, and by Høye et al. in six adults with cerebral palsy. Both studies showed the efficiency of the programs on study outcomes, and overall good feasibility (Isernia et al.; Høye et al.), suggesting that digital technology will play a crucial role in future neurorehabilitation models, with increased possibilities of customized care. Of course, there are limitations to neurorehabilitation and, for instance, Øra et al. found that internet connection issues have hindered telerehabilitation delivery in post-stroke aphasia. Digital technology has been used also to facilitate the remote assessment of clinical disability, patient's symptoms, adverse events, and outcomes. Sensory symptoms are generally considered difficult to evaluate, and, in an observational study, the Vibration Sensory Analyzer-3000 (VSA-3000) has shown higher diagnostic accuracy than the tuning fork in patients with impaired vibration sensation caused by central nervous system injury (stroke or spinal cord injury) (Gao et al.). Also, in another study, static post urography was able to detect subtle balance changes, and to discriminate healthy subjects from MS patients without clinically overt disability, thus suggesting this could be used to complement neurological examination for a more sensitive and objective assessment of balance and subsequent risk of falls (Inojosa et al.).

Advances in digital health and information technology have allowed collecting clinical data in a standardized and quantitative way, facilitating both research and patient care, especially in the MS field. For instance, the “Integrated Care Portal MS” is a portal for MS patients and health care professionals encompassing a pathway-based care model to better diagnose, monitor long-term, and thus optimally treat individual MS patients (Voigt et al.). Similarly, the “MS Documentation System” enabled the collection of clinical data into an eHealth platform (Ziemssen et al.), and the “MS Partners Advancing Technology and Health Solutions” allowed standardized data collection across 10 healthcare institutions (Mowry et al.). Moreover, Allen-Philbey et al. showed the potential of collecting data by combining clinical assessments and patient-reported outcomes, using a platform shared between a large data repository, the UK MS Register at Swansea University, and BartsMS in east London, UK. As such, authors have facilitated databases for research, service audit, and individual patient care, and have specifically highlighted the important role of public and patient involvement throughout the design and implementation process (Allen-Philbey et al.).

Shortcomings of digital technology could include its feasibility in the neurology field. However, Lavorgna et al. have investigated the attitude of neurologists toward the use of the internet, and showed a broad use of digital devices in clinical practice, with more than half participants using social media for communicating with patients, suggesting this is prime time for digital technology in neurology clinical practice.

In conclusion, this Research Topic has shown current applications of digital technology in neurology, from clinical assessment to data collection and rehabilitation. Results hereby presented have further gained relevance over the recent months, in light of the COVID-19 pandemic with many consultations and clinical assessments being now delivered remotely (3). In the future, based on these findings, we will be able to improve individualized care in neurological diseases, while keeping patients fully engaged in their management plan.

## Author Contributions

MM and FB: literature search and drafting the manuscript. SBr and SBo: literature search and revising the manuscript. All authors contributed to the article and approved the submitted version.

## Conflict of Interest

The authors declare that the research was conducted in the absence of any commercial or financial relationships that could be construed as a potential conflict of interest.
